# Abnormal skeletal and cardiac development, cardiomyopathy, muscle atrophy and cataracts in mice with a targeted disruption of the *Nov *(*Ccn3*) gene

**DOI:** 10.1186/1471-213X-8-18

**Published:** 2008-02-20

**Authors:** Emma Heath, Dalal Tahri, Elisabetta Andermarcher, Paul Schofield, Stewart Fleming, Catherine A Boulter

**Affiliations:** 1 Department of Genetics, University of Cambridge, Cambridge CB2 3EH, UK; 2 Department of Physiology, Development and Neuroscience, University of Cambridge CB2 3EG, UK; 3 Department of Pathology and Neuroscience, University of Dundee, Ninewells Hospital, Dundee DD1 9SY, UK

## Abstract

**Background:**

Signals from the extracellular environment control many aspects of cell behaviour including proliferation, survival, differentiation, adhesion and migration. It is increasingly evident that these signals can be modulated by a group of matricellular proteins called the CCN family. CCN proteins have multiple domains through which they regulate the activities of a variety of signalling molecules including TGFβ, BMPs and integrins, thereby influencing a wide range of processes in development and disease. Whilst the developmental roles of CCN1 and CCN2 have been elucidated, very little is known about the function of CCN3 (NOV). To investigate this, we have generated mice carrying a targeted mutation in the *Nov *gene (*Nov*^*del3*^) which reveal for the first time its diverse functions in embryos and adults.

**Results:**

By replacing *Nov *exon 3 with a TKneomycin cassette, we have generated *Nov*^*del3*^-/- mice which produce no full length NOV protein and express at a barely detectable level a mutant NOV protein that lacks the VWC domain. In *Nov*^*del3*^-/- embryos, and to a lesser extent in *Nov*^*del3*^+/- embryos, development of the appendicular and axial skeleton was affected with enlarged vertebrae, elongated long bones and digits, delayed ossification, increased bone mineralization and severe joint malformations. Primary embryo fibroblasts from *Nov*^*del3*^-/- mutant embryos showed enhanced chondrogenesis and osteogenesis. Cardiac development was also influenced leading to enlargement and abnormal modelling of the endocardial cushions, associated with septal defects and delayed fusion. In adults, cardiomyopathy was apparent, with hypertrophy and calcification of the septum and left ventricle dilation. Muscle atrophy was seen by 5 months of age, associated with transdifferentiation to fat. Premature tissue degeneration was also seen in the lens, with cataracts present from 6 months.

**Conclusion:**

We have generated the first mice with a mutation in the *Nov *gene (*Nov*^*del3*^). Our data demonstrate that NOV is a regulator of skeletal and cardiac development, and implicates NOV in various disease processes including cardiomyopathy, muscle atrophy and cataract formation. *Nov*^*del3 *^mutants represent a valuable resource for studying NOV's role in the modulation and co-ordination of multiple signalling pathways that underpin organogenesis and tissue homeostasis.

## Background

The behaviour of cells in development, tissue regeneration and disease is dependent upon multiple signals from the extracellular environment. These signals are mediated through a variety of signalling proteins that regulate cell proliferation, survival, differentiation, adhesion and migration. There is increasing evidence that a family of matricellular proteins, the CCN family, is a central player in regulating several of these signalling molecules (reviewed in [1]). By modulating their activities, CCN family members profoundly influence the behaviour of cells in development, wound healing, tissue homeostasis and in a range of diseases, including fibrosis and cancer.

There are six members of the CCN family: CCN1 (Cyr61), CCN2 (connective tissue growth factor, CTGF), CCN3 (Nov), CCN4 (WISP1), CCN5 (WISP2/rCOP-1) and CCN6 (WISP3) [2,3]. Their diverse effects are mediated by four cysteine-rich conserved domains which are shared by all members of the family, with the exception of WISP2 which lacks the C-terminal domain [2]. Through these domains, CCN proteins interact with a variety of extra-cellular signalling molecules, thereby regulating and potentially co-ordinating their activities. The first domain shares homology with insulin-like growth factor binding proteins (IGFBPs) and with Twisted gastrulation (Tsg) which modulates BMP signalling [4-6]. The second domain contains a Von Willebrand's factor type C repeat (VWC) and shares similarities with Short gastrulation (Sog)/Chordin; this domain in CTGF has been shown to enhance TGFβ binding to its receptor and inhibit BMP4 signalling [7]. The third domain contains a thrombospondin type 1 (TSP-1) repeat and in CTGF binds to the low-density lipoprotein (LDL) receptor-related protein 1 (LRP1) in a heparin-dependent manner [8,9]. The fourth carboxy- terminal (CT) domain is similar to the C terminus of Slit, which is involved in axon guidance and cell migration [10]. The CT domain mediates interactions with heparan sulphate proteoglycans [11,12] and contains a cysteine knot, a structure found in several growth factors, including TGFβ, platelet derived growth factor (PDGF) and nerve growth factors (NGFs) [2,13]. The CT domain of CTGF has been shown to interact with the Wnt co-receptor LDL receptor-related protein 6 (LRP6), thereby inhibiting Wnt signalling [14]; a similar domain is also present in another novel modulator of Wnt signalling, WISE [15] and modulation of Wnt signalling through this domain has also been demonstrated for Cyr61 [16]. Finally, many of the effects of different CCN family members involve signalling through a variety of integrins which interact specifically with different domains in the CCN proteins [17].

CCN proteins have a diverse range of activities in development and disease, and different family members mediate cell adhesion and migration, affect cell proliferation and survival, and influence angiogenesis, chondrogenesis and wound healing [18-23]. The involvement of two CCN proteins in regulating different aspects of development has been revealed by generating mutant knockout mice. Targeted disruption of CTGF identified a role in coordinating chondrogenesis and angiogenesis [24], while knock out mice lacking CYR61 show that it is required for placental development and vascular integrity [25]. Mutations in a third CCN gene, WISP3, cause progressive pseudorheumatoid dysplasia in man [26].

*Nov *was originally isolated from a chick nephroblastoma induced by infection of new born chicks with the MAV-1 avian retrovirus [27]. Nephroblastomas (Wilms' Tumours in humans) arise from the blastemal cells of the kidney and are characterised by abnormal proliferation and aberrant differentiation of this stem cell population. Whilst *Nov *is over-expressed in all nephroblastomas and Wilms' tumours studied [28,29] a direct causative link with *Nov *and tumour formation has only recently been demonstrated, with the isolation of a second independent proviral insertion in another virally-induced chick nephroblastoma [30]. The observation that *Nov *expression is deregulated in a variety of other tumour types, including musculoskeletal tumours [31] suggests that NOV may have a more general involvement in tumourigenesis.

The expression pattern of *Nov *during mammalian embryogenesis is consistent with a developmental role in a variety of tissues, including the cardiovascular system, skeleton, muscle and structures of the nervous system derived from the neural crest and placodes [32,33]. To investigate the function of NOV, we have generated mice carrying a targeted mutation in the *Nov *gene. Here we show that mutation of *Nov *leads to abnormal skeletal and cardiac development, to joint abnormalities, cardiomyopathy, and premature tissue degeneration causing muscle atrophy and cataracts in adult mice.

## Results

### Generation of mice carrying a targeted mutation in *Nov*

**Figure 1:**
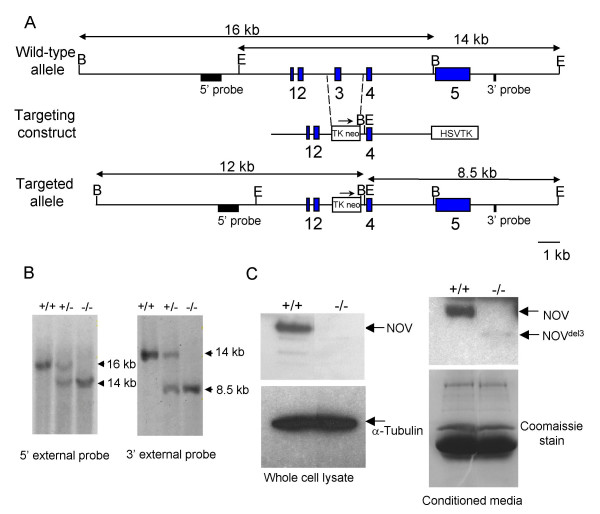
**Gene targeting at the *Nov *locus**. A. Schematic diagram showing wild type and targeted *Nov *alleles, the targeting construct, and the positions of the 5' and 3' external probes used to confirm targeting by Southern blotting. The TkneopolyA cassette replaces exon 3 in the targeted allele while the HSV TK cassette allows negative selection with Gancyclovir. B. Southern analysis of DNA from wild type, heterozygous and homozygous embryos using 5' and 3' external probes to confirm targeting. Digestion with *Bam*HI gave a 16 kb wild type band with the 5'external probe and 14 kb targeted allele with the 5' probe, while digestion with *Eco*RI gave a 14 kb wild type band and 8.5 kb targeted band with the 3' probe. C. Western blotting of whole cell lysates and conditioned media from *Nov*^*del3 *^homozygous and wild type E13.5 PEFs, using an anti-NOV antibody (59.3). Although NOV protein is readily detectable in wild type PEF whole cell lysates, no NOV protein can be detected in *Nov*^*del3 *^homozygous PEFs. Conditioned medium from wild type PEFs contains high levels of secreted NOV, whereas that from *Nov*^*del3*^-/- PEFs contains trace amounts of mutant NOV protein lacking the VWD domain.

*Nov*^*del3 *^mutant mice were generated by replacement of exon 3 with a Tkneomycin selection cassette (Figure 1A,B), resulting in the targeted allele encoding a protein that lacks the VWC domain. Both the wild type and targeted *Nov *alleles were expressed in primary embryonic fibroblasts (PEFs) derived respectively from wild type and *Nov*^*del3*^-/- E13.5 embryos (see Additional file 1). However, Western blotting showed that NOV protein was undetectable in whole cell lysates of *Nov*^*del3*^-/- PEFs, whereas it was expressed highly in PEFs from wild type littermates (Figure 1C). We were also unable to detect any full length NOV protein in conditioned medium from *Nov*^*del3*^-/- cells, but we could detect trace amounts of the mutant protein lacking the VWC domain. This was present at an extremely low level compared to that of the full length NOV protein in conditioned medium from wild-type PEFs, suggesting that deletion of the VWC domain might lead to reduced protein stability (Figure 1C).

Analysis of genotype ratios of offspring of F1 and F2 heterozygous matings indicated that there was loss of 35% of heterozygotes and 55% of homozygotes prior to post natal day10, with loss of 10% heterozygous and 20% homozygous embryos isolated at E13.5-E19.5 (Table 1). Surviving *Nov*^*del3*^+/- and *Nov*^*del3*^-/- littermates were in apparent good health, and as adults were both viable and fertile. Successive generations showed reduced levels of neonatal loss, possibly reflecting selection for a less severe phenotype.

**Table 1:** Genotypes of Offspring from Matings of F1 and F2 *Nov*^*del3 *^heterozygotes

	Number of litters	*Nov*^*del3 *^-/-	Wild type	*Nov*^*del3*^+/-
Embryos E12.5-E19.5	15	22	28	50
Ratio: wild type		0.8	1.0	1.8
% Loss		20	0	10
Live born >P10	7	5	11	14
Ratio: wild type		0.45	1.0	1.3
% Loss		55	0	35

### Skeletal development is affected in *Nov*^*del3 *^homozygotes and heterozygotes

**Figure 2:**
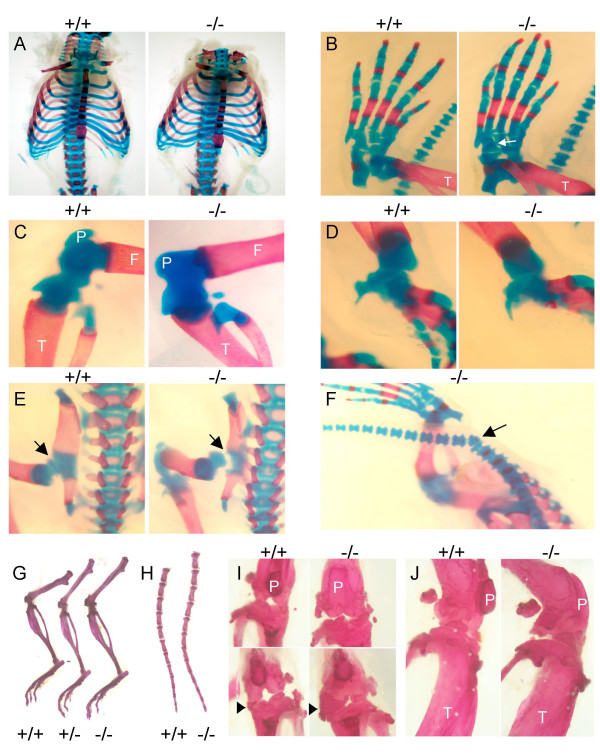
**Skeletal staining of wild type and *Nov*^*del3*^-/- E19.5 embryos and adults with Alcian blue (cartilage) and Alizarin red (bone)**. A-F: *Nov*^*del3*^-/- E19.5 embryos. A. Staining of rib cages showing barrel chest in *Nov*^*del3 *^-/- with overgrowth of ribs. B. Hind foot showing fusion of the tarsal cartilage elements (white arrow) and elongation of the digits in *Nov*^*del3 *^-/-. Note increased intensity of Alizarin red staining and thickening of the tibia in *Nov*^*del3*^-/- compared to wild type. C. Knee abnormalities in *Nov*^*del3*^-/- compared with wild type, including flattening of the patella. D. Malformation of the wrist elements in *Nov*^*del3 *^-/- compared to wild type. E. Dislocation of hip (arrow) in *Nov*^*del3*^-/- compared to wild type. F. Kinking of tail with compression of the vertebral body (arrow) in *Nov*^*del3 *^-/- embryo. G-J: Adult skeletons. Overgrowth of the appendicular skeleton in *Nov*^*del3 *^-/-, and to a lesser extent in *Nov*^*del3 *^+/-, compared to wild type (+/+) littermate. H. Overgrowth of the axial skeleton in *Nov*^*del3*^-/- compared to wild type, with increased length of individual vertebral bodies. I. Frontal view of right (upper panel) and left (lower panel) knee joints from *Nov*^*del3*^-/- and wild type (+/+) littermates showing abnormal patella (P) and grossly enlarged medial meniscus (arrow head) in mutant compared with wild type. J. Lateral view of knee showing flattening of patella in *Nov*^*del3*^-/-. F: femur; T: tibia and P: patella.

Analysis of skeletons isolated from embryos at late gestation (E18.5 and E19.5) indicated abnormalities in *Nov*^*del3*^-/- embryos (n = 6/6) and, with lesser severity, in *Nov*^*del3*^+/- embryos (n = 8/8), compared to wild type embryos (n = 0/3). Mutant skeletons were larger than those of wild type littermates and showed evidence of overgrowth of skeletal elements with enlarged vertebrae, elongated long bones and digits (Figure 2). Heterozygous and homozygous mutants had barrel-chested rib cages which might have contributed to the neonatal deaths observed (Figure 2A). Joint defects were also apparent, including fusion of the tarsal bones in the foot (Figure 2B), flattening of the patella (Figure 2C), malformation of the wrist (Figure 2D), dislocation of the hip (Figure 2E) and abnormal articulation of the joints resulting in laxity of the limbs. Abnormalities in segmentation of the caudal vertebrae were also observed in some animals, causing kinking of the tail (Figure 2F).

These skeletal abnormalities were also manifest in adult homozygotes, and to a lesser extent in adult heterozygotes, on staining skeletal preparations with Alcian blue and Alazarin red. In the mutants, but not in wild types, we observed overgrowth of the appendicular and axial skeleton, with increased length of the long bones (Figure 2G) and enlarged vertebrae (Figure 2H) (*Nov*^*del3*^-/- n = 6/6; *Nov*^*del3*^+/- n = 4/6; wild type n = 0/5). Joint abnormalities were also seen with knee deformities being particularly prominent, characterised by expansion of the meniscus (Figure 2I) and abnormalities of the articular surfaces (Figure 2I,J) (*Nov*^*del3*^-/- n = 6/6; *Nov*^*del3 *^+/- n = 4/6; wild type n = 0/5). Compared to wild type littermates, the skulls of heterozygotes and homozygotes were slightly larger and flatter (data not shown).

### Abnormal chondrocyte differentiation

**Figure 3:**
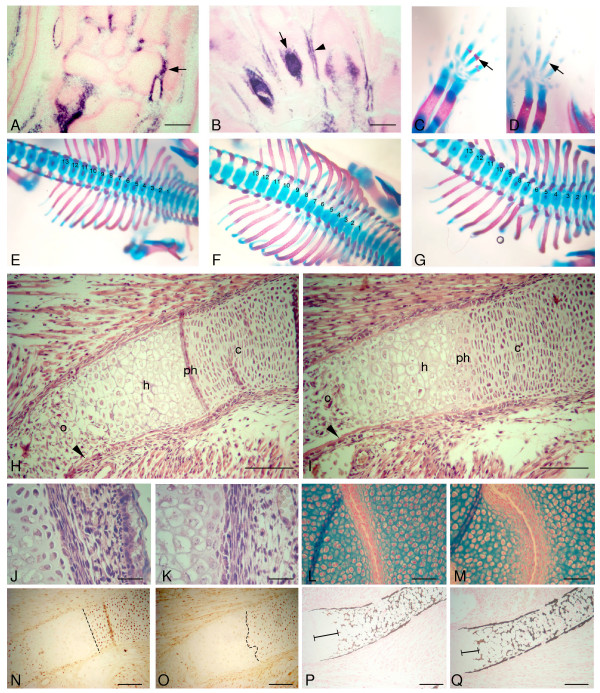
***Nov *expression and phenotypes of E16.5 skeletons**. A and B. RNA *in situ *hybridization showing *Nov *expression in the hind foot at E16.5. A. Strong expression in the myotendenous junctions (arrow) and in the mesenchyme of the joints adjacent to the cartilage elements, but no expression in condensing cartilage. B. High expression in the mesenchyme overlying the cartilage elements (arrow) and in the digital tendons (arrow head). C-G. Skeletal staining with Alizarin red and Alcian blue of E16.5 *Nov*^*del3*^+/-, *Nov*^*del3*^-/- and wild type (+/+) embryos. C. Wild type forefoot showing ossification of digits (arrow). D. No ossification in *Nov*^*del3*^-/- digits (arrow), but intense staining with Alizarin red of the radius and ulna indicating increased bone mineralization. E-G Vertebrae and rib cages of wild type (E), *Nov*^*del3*^-/- (F) and *Nov*^*del3*^+/- (G) showing delay in ossification of the vertebrae in the mutant embryos. No ossification is present in the *Nov*^*del3*^-/- embryo, and whilst ossification is taking place in the *Nov*^*del3*^+/- embryo (G), it is not occurring in the ordered, sequential manner observed in the wild type. Thoracic vertebrae numbered 1–13. H-Q. Histological sections of E16.5 wild type and *Nov*^*del3*^-/- embryos. H, J, L, N, P: wild type; I, K, M, O, Q: *Nov*^*del3*^-/- littermate. H, I: Haematoxylin and Eosin stained sections showing expanded perichondrium and periosteum in *Nov*^*del3*^-/- and a thicker bone collar (arrow head) compared to the wild type. o: ossified bone; h: hypertrophic cartilage; ph: pre-hypertrophic cartilage; c: columnar chondroctyes. J, K: Haematoxylin and Eosin stained sections at higher magnification at the junction of pre-hypertrophic/hypertrophic chondrocytes, adjacent to the border of the perichondrium/periosteum, showing abnormal morphology of chondrocytes and matrix in the mutant (K). L, M: Alcian blue staining of cartilage, showing expansion of the cartilage element and blurring of its borders in the *Nov*^*del3*^-/- embryo (M) compared to wild type (L). N, O: PCNA staining showing a sharp demarcation (black dashed line) between proliferating chondrocytes and pre-hypertrophic chondroctes in the wild type (N), but not in the *Nov*^*del3*^-/- mutant (black dashed line) (O). P, Q: Von Kossa staining for mineralised bone showing a shorter and thicker bone collar (indicated by the black bar) in the pre-hypertrophic/hypertrophoic zone of the *Nov*^*del3*^-/- mutant (Q) compared to wild type (P). Scale bars in A B = 20 μm; H,I,N,O,P,Q = 10 μm; J-M = 5 μm.

The expression pattern of *Nov *is consistent with a role in skeletal development. We have shown that it is expressed in the mesenchyme surrounding cartilage condensations, and in the tendons and myotendenous junctions at E16.5 in the mouse (Figure 3A,B; [32]), while others have reported expression in pre-hypertrophic and hypertrophic cartilage [34]. To investigate the origin of the skeletal abnormalities, skeletal preparations were made from embryos at E16.5. Alcian blue staining indicated that the cartilage elements were already enlarged in the *Nov*^*del3*^-/- (n = 2/2) and *Nov*^*del3*^+/- embryos (n = 4/4), while Alizarin red staining of bone indicated a delay in ossification of the digits and vertebrae in the mutants (n = 5/6) compared to their wild type littermates (n = 0/3) (Figure 3C–G). The tightly ordered sequential ossification of the vertebrae seen in wild type embryos was disrupted in the mutants, indicating a defect in the normal differentiation of cartilage to bone (Figure 3E–G). However, once ossification did occur the intensity of Alizarin red staining was greater in the homozygotes (n = 2/2) and heterozygotes (n = 4/4) than in wild type embryos (n = 0/3), suggesting increased bone mineralization (Figure 3C,D). This was also observed in mutant embryos at E18.5 (data not shown) and E19.5 (Figure 2A–F).

Histological analysis of *Nov*^*del3*^-/- E16.5 embryos revealed abnormalities in chondrogenesis (n = 4/4) which were not apparent in wild type littermates (n = 0/4). Haematoxylin and Eosin stained sections from *Nov*^*del3*^-/- embryos showed morphological differences in the pre-hypertrophic and hypertrophic cartilage and their surrounding matrix (Figure 3J,K,L,M). The chondrocytes adjacent to the perichondrium near the junction with the periosteum were enlarged compared with chondrocytes from wild type embryos (Figure 3J,K), with increased thickness of the perichondrium and periosteum compared to wild type littermates (Figure 3H–M). Alcian blue staining confirmed increased size of the cartilage elements in the *Nov*^*del3 *^-/- embryos and indicated blurring of their boundaries, showing that the demarcation between mesenchyme and chondrocytes seen in the wild type embryos was absent in the mutants, and suggesting an expansion of the chondrocyte domain (Figure 3L,M). On staining with an antibody against the proliferation marker PCNA the sharp junction of proliferating columnar chondrocytes with non proliferating pre-hypertrophic chondrocytes seen in wild type sections was absent in the homozygous mutants, suggesting that the ordered transition from the columnar to pre-hypertrophic state was disrupted (Figure 3N,O). Von Kossa staining for mineralized bone also confirmed a shortening of the bone collar in the pre-hypertrophic/hypertrophic region (n = 4/4) compared with the wild type control (n = 0/4), consistent with a reduction in the size of this zone, while the bone collar itself was thicker and stained more intensely, indicating a greater quantity of mineralized bone matrix (Figure 3P,Q).

### Enhanced chondrogenesis and osteogenesis of *Nov*^*del3*^-/- fibroblasts

We hypothesised that the enlarged cartilage elements and increased bone mineralization seen in *Nov*^*del3*^-/- embryos might reflect increased chondrogenesis and osteogenesis. We therefore determined whether primary embryo fibroblasts (PEFs) from *Nov*^*del3*^-/- embryos showed enhanced participation in chondrogenesis and/or osteogenesis.

**Figure 4:**
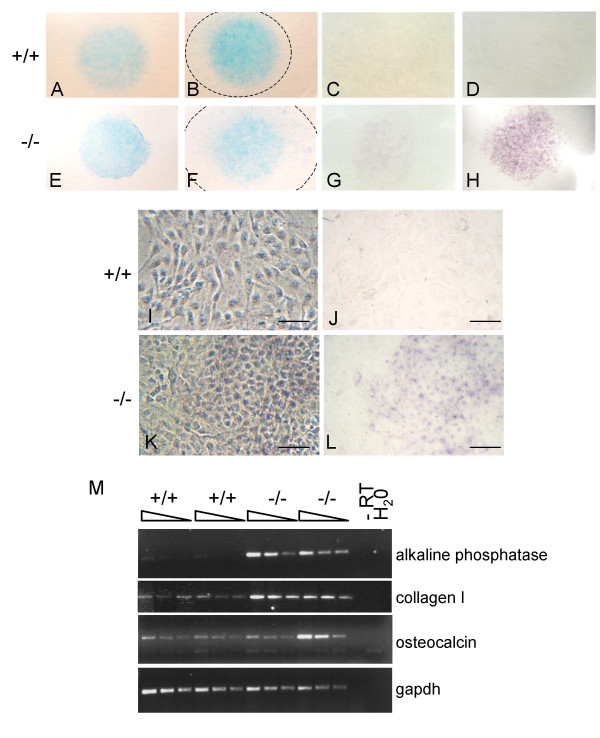
**Enhanced chondrogenesis and osteogenesis in *Nov*^*del3*^-/- PEFs**. A-H: Staining of micromass cultures of PEFs derived from wild type (A-D) and *Nov*^*del3*^-/- (E-H) embryos. Alcian blue staining after 5 days (A, E) and 9 days (B, F) in culture showing enhanced cartilage differentiation in *Nov*^*del3*^-/- cells, with the dashed line in B and F marking the extent of the micromass. Alkaline phosphatase staining after 5 days (C, G) and 9 days (D, H) in culture showing enhanced osteoblast differentiation in *Nov*^*del3*^-/- cells. I-L: Morphology of monolayer PEF cultures derived from wild type (I, J) and *Nov*^*del3*^-/- (K,L) embryos with (I, K) and without (J, L) phase contrast. Alkaline phosphatase staining after 10 days in culture (I, J, K, L) showing osteoblast differentiation in the *Nov*^*del3*^-/- PEFs but not in the wild type PEFs. Scale bar in I-N = 10 μm. M: Semi quantitative RT-PCR of mRNA from wild type (+/+) and *Nov*^*del3*^-/- PEFs using primers for alkaline phosphatase, collagen I and osteocalcin. Two fold serial dilutions of cDNA were used and normalised to *gapdh*.

Three independent wild type PEF cell lines and three *Nov*^*del3 *^homozygote cell lines were isolated from individual E13.5 embryos and explanted as micromasses. After culturing for 5 and 9 days, the micromasses were stained with Alcian blue as a marker of chondrogenesis and alkaline phosphatase for osteogenesis. After 5 days, the *Nov*^*del3*^-/- micromasses stained more intensely with Alcian blue than did the controls (Figure 4A,E) and by 9 days had increased significantly in diameter compared to wild type micromasses (Figure 4B,F). Osteogenic differentiation was also observed in the *Nov*^*del3*^-/- micromasses: weak alkaline phosphatase activity was detected in the *Nov*^*del3*^-/- micromasses at day 5, with strong staining at day 9, in contrast to control cultures which were negative at both time points. Hence, both chondrogenesis and osteogenesis were enhanced in the *Nov*^*del3*^-/- micromasses.

We next investigated whether the osteogenesis seen in the *Nov*^*del3*^-/- micromass cultures was a result of an increased tendency of *Nov*^*del3*^-/- mesenchymal cells to differentiate down this pathway. Three independent *Nov*^*del3*^-/- cell lines and three control wild type cell lines were plated at passage 2 and cultured for 10 days in DMEM with 10% FCS, after which they were stained for the osteoblast marker alkaline phosphatase. In contrast to the control cultures which retained a fibroblast phenotype and were negative for the osteoblast marker (Figure 4I,J), the Nov^*del3 *^-/- cultures showed extensive staining (Figure 4K,L). RTPCR performed on two independent wild type and homozygous cultures showed high expression of the osteoblast markers alkaline phosphatase and collagen I in the *Nov*^*del3*^-/- cultures, but not in the wild type cell lines, with one of the mutant cell lines exhibiting increased expression of the later osteogenic marker osteocalcin (Figure 4M).

### Abnormal development, cardiomyopathy and calcification of *Nov*^*del3 *^mutant hearts

**Figure 5:**
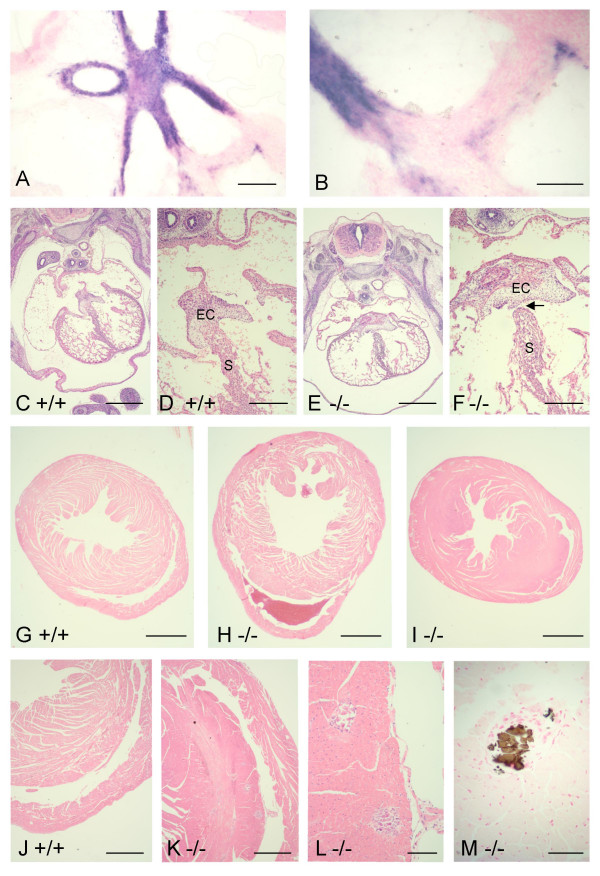
***Nov *expression and histology of embryonic and adult hearts**. A, B: RNA *in situ *hybridization showing *Nov *expression in the endothelial and smooth muscle cells of the aortic outflow tract, pulmonary trunk and in a subset of cells near the origins of the great vessels at E16.5. C-F: Haematoxylin and Eosin stained sections of wild type (C,D) and *Nov*^*del3*^-/- (E,F) E13.5 embryonic hearts showing abnormal expansion of the endocardial cushions (EC) and delay in fusion of the septum (S) in the mutant mouse (arrow). G-M: Haematoxylin and Eosin stained sections of wild type (G, J) and *Nov*^*del3*^-/- (H, I, K) adult hearts, showing accumulation of blood in the sub-endothelial space between the right ventricle and septum (H) and hypertrophy of the septum near the origins of the great vessels, (H, I, K). Areas of calcification on the septal wall of the right ventricle (K, L), stain with Von Kossa (M). There is no associated fibrosis. Scale bars in A,D,F = 20 μm; B,L = 10 μm; C,E = 50 μm; G,H,I = 1 mm; J,K = 50 μm; M = 5 μm.

During mouse heart development, *Nov *is expressed highly in the endothelial and smooth muscle cells of the aortic outflow tract from E12.5 and in a subset of cells near the origins of the great vessels (Figure 5A,B; [32]). Defects in heart development were observed in E13.5 *Nov*^*del3*^-/- embryos. Haematoxylin and eosin stained transverse serial sections showed abnormal growth and modelling of the endocardial cushions (n = 4/4); these were enlarged and exhibited a broader base extending laterally compared to wild type littermates (n = 0/3) (Figure 5C–F; Additional file 2). Abnormalities in development of the septum were also seen, namely thickening of the septum and a delay in fusion with the endocardial cushions (Figure 5D,F). No heart defects were observed in wild type littermates (n = 0/3). Serial transverse sectioning of adult hearts showed that *Nov*^*del3*^-/- mice (n = 3/3), and to a lesser extent *Nov*^*del3*^+/- mice (n = 3/3), exhibited defects at the caudal end of the septum near the origins of the great vessels, including hypertrophy of the septum towards the right ventricle and accumulation of blood in the sub-endothelial space between the right ventricle and septum; these defects were not observed in wild type littermates (n = 0/3) (Figure 5G–K). Hypertrophic cardiomyopathy of the right and left ventricles and ventricular dilation suggestive of cardiac failure were noted in both homozygous and heterozygous *Nov *mutants, but not wild types (Figure 5G–K). Staining with Von Kossa indicated that areas of calcification were also present, most markedly on the septal wall of the right ventricle (Figure 5K–M). Deposition of calcium was within morphologically normal myocardial tissue and there was no evidence of fibrous scarring, but occasionally a giant cell response to the calcium deposit was noticed. Venous dilation and congestion was observed in the kidney, liver and other organs, but the major arteries appeared normal.

### Tissue degeneration in adult *Nov*^*del3 *^homozygotes and heterozygotes

**Figure 6:**
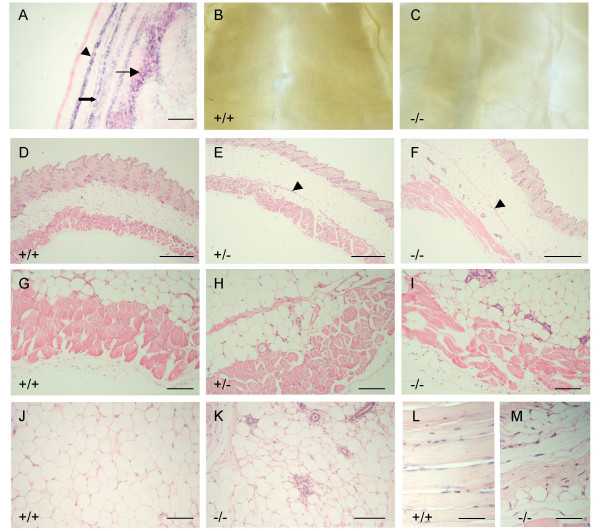
***Nov *expression and phenotype of subcutaneous and hypaxial muscles**. A: RNA *in situ *hybridization showing *Nov *expression in subcutaneous (arrow head), body wall (filled arrow) and intervertebral muscles (arrow) in E16.5 embryos. B, C: photographs of wild type (B) and *Nov*^*del3*^-/- (C) body walls, showing thinning and transparency in the mutant. D-I: Haematoxylin and Eosin stained sections of adult skin from wild type (D, G), *Nov*^*del3*^+/- (E, H) and *Nov*^*del3*^-/- (F, I) mice showing atrophy of subcutaneous muscles in *Nov*^*del3*^+/- and *Nov*^*del3*^-/- mice, with arrow heads marking the residual muscle layer. Histology of the subcutaneous adipose tissue in wild type (J) and *Nov*^*del3*^-/- (K) showing large mature fat cells in the wild type in contrast to the mixture of large (mature) and small (immature) fat cells in the mutant. L, M: Haematoxylin and Eosin stained section of adult intercostal muscles in wild type (L) and *Nov*^*del3*^-/- (M) mice showing muscle atrophy and transdifferentiation to fat in the mutant. Scale bars in A = 20 μm; D-F = 50 μm; G-M = 10 μm.

The sites of *Nov *expression during mouse muscle development are in the lateral dermomyotome, in the subcutaneous muscle and in a subset of hypaxial muscles, notably the hip, shoulder, body wall, inter-costal and inter-vertebral muscles, but not muscles of the limb (Figure 6A; [32]). We noted that the body walls of *Nov*^*del3*^ homozygous adults (n = 11/11), and to a lesser extent heterozygotes (n = 19/20), were abnormally thin and more transparent than those from wild type littermates (n = 0/15) (Figure 6B,C). Other muscles that normally express *Nov *were severely affected in both adult homozygotes and to a lesser extent heterozygotes. From five months of age, these muscles exhibited areas of degeneration characterised by atrophy and trans-differentiation to immature adipose cells. In the subcutaneous muscles the extent of muscle atrophy could be clearly observed in the mutants, with a very thin line of residual muscle cells demarcating the normal junction of the subcutaneous fat and muscle layers; no atrophy was seen in their wild type littermates (Figure 6D–I). The cells in the immature adipose tissue had a characteristic bubbly cytoplasmic morphology and small size, in contrast to the large regular mature adipose cells present in the normal subcutaneous adipose tissue (Figure 6J,K). The process of muscle atrophy was clearly observed in the intercostal muscles with trans-differentiation to fat occurring within the muscle tissue (Figure 6L).

**Figure 7:**
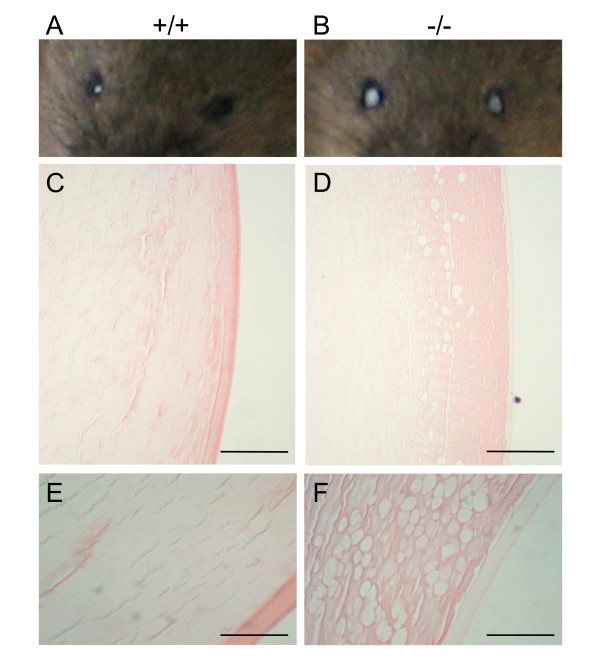
**Phenotype of adult lens**. A, B: Photograph of eyes of wild type (A) and *Nov*^*del3*^-/- (B) adult mice of six months of age, showing cataracts in both eyes in the mutant. The small white colouration in the wild type eye is an artefact due to reflected light. C-F: Haematoxylin and Eosin stained sections of wild type (C,E) and *Nov*^*del3*^-/- (D,F) adult eyes showing degeneration of the mutant lens, with vacuolation and loss of surface epithelium. Scale bars in E,F = 5 μm; C,D = 15 μm.

Early onset tissue degeneration was also seen in the lens with cataracts present in the eyes of both *Nov*^*del3*^-/- (n = 10/23) and *Nov*^*del3*^+/- (n = 9/28) mice between six and thirteen months of age but not in wild type mice of similar age (n = 0/14) (Fig 7A,B). Haematoxylin and Eosin stained sections of wild type and homozygous adult eyes showed vacuolation of the degenerating lenses in the mutant with loss of the surface epithelium and fragmentation of the abnormal lens tissue (Figure 7C–F).

## Discussion

We have generated by gene targeting *Nov*^*del3*^-/- mice that reveal diverse roles of NOV in the developing embryo and in tissue maintenance in adult mice. These mice produce no full length NOV protein, but express at a barely detectable level a mutant NOV protein that lacks the VWC domain. The low level of the mutant protein was in contrast to the expression level of its mRNA, which in PEFs was comparable to that of the wild type transcript, suggesting that deletion of the VWC domain might lead to reduced NOV protein stability.

Interestingly, we have detected by RT-PCR an equivalent *Nov* transcript also lacking the VWC domain in several tissues in the developing wild type mouse embryo at E16.5 (DT, CB unpublished observations). Sequence analysis of the RT-PCR product confirms that this is a naturally occurring splice variant lacking exon 3 and hence the VWC domain. We note that a splice variant of WISP1 similarly lacking the VWC domain has also been reported [35]. In CTGF, the VWC domain has been shown to enhance TGFβ binding to its receptor and inhibit BMP4 signalling [7]. It is therefore possible that variant NOV and WISP1 proteins lacking this domain may therefore be functionally distinct from full length NOV and WISP1.

On the basis of the analysis of *Nov*^*del3*^-/- mice, we cannot conclude whether the phenotypes observed are due to the loss of full length NOV function, or to possible novel functions of the very low level of NOV^del3 ^produced. The diverse range of phenotypes of *Nov*^*del3*^ homozygotes was also seen, albeit to a less severe extent, in *Nov*^*del3 *^heterozygotes. This would be consistent either with haploinsufficiency of the single remaining intact *Nov *gene, or alternatively that a low level of NOV^del3 ^mutant protein has a dominant effect. Indeed, preliminary experiments suggest that forced expression of *Nov*^*del3 *^in 10T1/2 fibroblasts affects their differentiation, suggesting that it can indeed have biological activity, although differences in the expression level, in cell types and developmental context involved mean that we cannot conclude whether this protein contributes to the *Nov*^*del3*^-/- *in vivo *phenotype. Resolution of this point will require generation of mice that are homozygous for a true null mutation in the *Nov *gene, and to date we have been unsuccessful in achieving this, for reasons unknown. Hence, in the absence of the NOV null mutant, it is not possible to determine whether the phenotypes are due to loss or reduction in the level of full length NOV, the NOV^del3 ^mutant protein or a combination of both.

The phenotypes seen in the *Nov*^*del3 *^mutant mice show both significant similarities and differences with knockouts of other CCN genes, discussed in more detail in later sections. Phenotypes were observed in both *Nov*^*del3 *^heterozygotes and homozygotes, affected a variety of different tissues and resulted in embryonic or neonatal lethality of a subset of offspring. In these respects, the *Nov *mutant differs from the reported *Cyr61 *and *Ctgf *knockout mice, both of which are null mutants with lethality of all homozygotes in utero (*Cyr61*) or shortly after birth (*Ctgf*), although we cannot conclude whether the NOV^del3 ^protein is sufficient to rescue an otherwise embryonic lethality. While *Nov *is expressed in the smooth muscle and endothelial cells of the major vessels, no severe abnormalities were observed in these tissues, in contrast to the *Cyr61 *knockout homozygotes which die in mid-gestation with placental defects and loss of vascular integrity [25]. However, *Nov*^*del3*^-/- embryos did exhibit abnormalities in the endocardial cushions and delay in septal fusion which has also been found in *Cyr 61*-/- embryos [36]. As in the *Ctgf *knockout homozygotes, many *Nov*^*del3*^-/- offspring had abnormalities in skeletal development, but with significant differences in phenotypes, as discussed below. Joint abnormalities were also observed in the *Nov*^*del3*^ mutants both before and after birth; this has not been reported for the *Ctgf *knockout. Mutations in another CCN family member, WISP3, cause progressive pseudorheumatoid dysplasia in man [26], although the mouse knockout in *Wisp3 *had no overt phenotype, suggesting that its loss in the mouse can be compensated for by another gene.

### Abnormal skeletogenesis and joint formation in *Nov*^*del3 *^mutant mice

*Nov*^*del3*^+/- and *Nov*^*del3*^-/- mice exhibited multiple defects in skeletogenesis and joint formation. Overgrowth of the appendicular and axial skeleton was observed, with enlargement of vertebrae, long bones and digits, and fusion of tarsal bones in the foot. The cartilage elements were expanded in the *Nov*^*del3*^-/- embryos compared to wild type littermates, seen both in whole skeletal preparations at E16.5, E18.5 (data not shown) and E19.5 and in sectioned embryos at E16.5. Enhanced chondrogenesis was also seen in micromass cultures *in vitro*, with stronger staining with Alcian blue after 5 days in culture and increased diameters of the micromasses at 9 days. It will be of interest to determine whether this reflects increased proliferation and/or migration of chondrocytes and/or enhanced differentiation of mesenchymal cells down the chondrogenic pathway. Terminal chondrogenic differentiation in *Nov*^*del3*^-/- embryos was also affected; the morphology of the pre-hypertrophic cells and surrounding matrix was abnormal and ossification of hypertrophic cartilage was delayed, with a shortening of the pre-hypertrophic/hypertrophic zone and disruption of the sharp transition between proliferative columnar cells and quiescent pre-hypertrophic cells present in wild type embryos. Yu *et al*. [34], have reported that NOV is expressed in pre-hypertrophic and early hypertrophic chondrocytes, and that down-regulation of NOV expression by administration of parathyroid hormone-related protein (PTHrP), coincides with a delay in terminal differentiation. Lafont *et al*. [37], showed that administration of exogenous NOV up-regulated TGFβ2 and Collagen X, a marker of hypertrophic chondrocytes, suggesting that NOV acts as a promoter of late chondrocyte differentiation. A detailed molecular analysis of chondrogenic differentiation in the *Nov*^*del3*^-/- embryos will shed further light on the role of NOV in this pathway.

Although there was a delay in ossification of hypertrophic chondrocytes, an overall increase in the thickness of the bone collar and in the intensity of staining of the bone matrix with Von Kossa was observed in the *Nov*^*del3*^-/- embryos. The increased mineralization observed in the mutants may reflect enhanced differentiation down the osteogenic pathway, as the differentiation of mesenchymal cells to osteoblasts was promoted in PEFs and in micromasses derived from *Nov*^*del3*^-/- embryos. Our results are consistent with recent data from Rydziel and co-workers showing that over-expression of *Nov *inhibits osteoblastogenesis and causes osteopenia in transgenic mice expressing *Nov *from the osteocalcin promoter [38]. These authors show convincingly that the inhibition of osteogenic differentiation is achieved through NOV directly binding and inhibiting the activity of BMP2, a key regulator of skeletogenesis. They also showed that NOV has Wnt3/β-catenin antagonistic activity, but this was not via a direct interaction with Wnt3 or its co-receptor LRP-6. The increased bone mineralization we observed in the *Nov*^*del3*^-/- and *Nov*^*del3*^+/- mice is also consistent with the decrease in mineral apposition rate seen in the *Nov *transgenic mice described by Rydziel *et al*. which they ascribe to impaired osteoblastic function. Minamizato *et al*. [39] also reported that over-expression of *Nov *blocked osteogenic differentiation by interacting with and inhibiting BMP2, and also by activating Notch signalling; this conflicts with the results of Rydziel *et al*. who found that NOV inhibited Notch signalling in osteoblastic cells.

Our results differ significantly from those seen in CTGF knockout mice which die shortly after birth with severely malformed rib cages. As in the *Nov*^*del3 *^mutants, delayed ossification was also observed, but in this case the pre-hypertrophic/hypertrophic zone was enlarged and the *Ctgf *-/- mice exhibited thinner bone collars. In contrast, in the *Nov*^*del3 *^mutants, the size of the prehypertrophic/hypertrophic zone was reduced, and the bone collars were increased in thickness. In the *Ctgf*-/- mice, the delay in ossification reflects a requirement for CTGF in the coordination of ossification and angiogenesis. We have not studied angiogenesis in the *Nov*^*del3*^-/- mice, but it is known that NOV can act as an angiogenic factor [40], and this might therefore also contribute to the delay in ossification seen in the *Nov *mutants. The differences seen in the skeletal phenotypes of the *Ctgf *and *Nov *mutants may reflect the different roles played by these proteins in osteoblast differentiation. In a cell culture system in which mesenchymal stem cells were induced to differentiate down the osteogenic pathway on induction with Wnt3A, *Ctgf*, together with *Cyr61 *and *Wisp2 *were significantly upregulated, whereas *Nov *was not [41]. In this system, Cyr61 was a direct target of Wnt/β catenin signalling and RNA interference-mediated knockdown of Cyr61 reduced Wnt3A induced osteogenic differentiation. This contrasts markedly with the effects of NOV on inhibiting the osteogenic differentiation of ST-2 stromal cells and MC3T3 osteoblastic cells [38], which is mediated by direct inhibition of BMP2 and indirect inhibition of Wnt/β catenin signalling. The increased bone collar thickness in the *Nov*^*del3 *^-/- embryos would be consistent with an increase in Wnt3 signalling, as activating mutations in the Wnt receptor LRP5 cause high bone density in man [42,43] while loss of function mutations in this gene cause the reduced bone density seen in the autosomal recessive disorder osteoporosis-pseudoglioma syndrome [44]. It will be of interest to determine whether Wnt3/β catenin signalling is increased in the skeletal system of *Nov*^*del3*^ mutants. Further investigation into the mechanisms underlying the modifications in bone formation and remodelling in *Nov*^*del3*^mutant mice is currently underway.

Unlike *Ctgf*, *Nov *is very highly expressed in the myotendenous junctions during skeletal development, and abnormalities in joint formation were seen in both *Nov*^*del3*^+/- and *Nov*^*del3*^-/- mice. In adult mutant mice, knee deformities were particularly prominent, characterised by expansion of the meniscus and abnormalities of the articular surfaces. Mutations in *WISP3 *(CCN6) are associated with joint abnormalities in man, causing progressive pseudorheumatoid dysplasia, characterised by juvenile-onset cartilage degeneration [26], although *Wisp3 *knockout mice have no overt phenotype [45]. Our results suggest a possible involvement for NOV in diseases of the joints and further support the idea that multiple members of the CCN family are required for normal formation and homeostasis of the skeleton.

### Nov affects heart development and causes cardiomyopathy in adult mice

Our results show for the first time a requirement for NOV in heart development. *Nov *is expressed highly in the smooth muscle and endothelial cells of the major vessels, including the aorta and pulmonary trunk, and in a subset of cells near the base of the great vessels [32,33]. Although the major vessels appeared overtly normal in the *Nov*^*del3*^-/- embryos, abnormalities in the growth and modelling of the endocardial cushions were seen and a delay in fusion of the ventricular septum was noted. *Nov*^*del3*^-/- and *Nov*^*del3 *^+/- adults developed cardiomyopathy characterised by hypertrophy of the septal wall and calcification. Similar defects are also seen in knockout mice lacking the gap junction protein Connexin 43 (Cx43) [46]. Homozygous *Cx43 *knockout mice die shortly after birth with malformation of the conotruncal heart segment leading to ventricular outflow obstruction; these mice also exhibit hypertrophy of the septum and calcification. Interestingly, NOV has been shown to interact directly with Cx43 [47] and *Cx43 *null homozygotes also exhibit delayed ossification and osteoblast dysfunction. Skeletal and cardiovascular defects are also a feature of knockout mice with disrupted BMP signalling [48]. BMP4 is expressed in the outflow tract (OFT) myocardium and in the endocardial cushions from E12-E14 in the mouse while BMP2 is expressed in the atrioventricular canal and valves [49]. By generating knockout mice with a mutated BMP type II receptor with reduced signalling capability, Delot et al. [50], have shown that BMP signalling is required for growth of the OFT cushions, OFT septation and formation of the semilunar valves. The demonstration that NOV binds directly to BMPs and inhibits their activity in osteogenesis [38] raises the possibility that abnormalities in endocardial cushion development and ventricular septation of *Nov*^*del3 *^mutants may be mediated through alterations in BMP signalling.

A requirement for another CCN family member, CYR61, in development of the endocardial cushions has recently been reported [36]. *Cyr61*-/- embryos exhibit severe atrioventricular septal defects (AVSD) as a result of abnormal valvuloseptal morphogenesis. Interestingly, delayed formation of the ventricular septum is also seen in *Cyr61 *+/- embryos, and approximately 20% of *Cyr61 *+/- adults have persistent ostium primum atrial septal defects (ASD). Thus, haploinsufficency for *Cyr61 *causes cardiac defects, indicating that its level of expression is critical for normal heart development. We also see cardiac defects in adult *Nov*^*del3 *^heterozygotes, as well as abnormalities in other tissues, which would be consistent with haploinsufficency for *Nov*, although it is also possible that these phenotypes could be due to a dominant effect of the NOV^del3 ^mutant protein.

### Muscle atrophy and transdifferentiation of myocytes to adipocytes in *Nov*^*del3 *^homozygotes and heterozygotes

*Nov *is expressed highly in specific muscles during mouse development, notably the subcutaneous muscles, and a subset of hypaxial muscles: body wall, intercostal, intervertebral, hip and shoulder muscles [32]. All of these muscles developed in *Nov*^*del3*^-/- and *Nov*^*del3*^+/- embryos, but underwent premature degeneration by five months of age in the adult. Muscle atrophy was associated with transdifferentiation to fat, with the characteristic morphology of immature adipocytes. Thus, NOV is required for muscle maintenance and viability. It will be of interest to determine whether the satellite cells, which are the stem cell population involved in muscle regeneration, are normal in these mice, as we saw little evidence of muscle regeneration taking place in the atrophied areas.

There is increasing evidence that NOV is a key regulator of myogenesis. *In vitro*, *Nov *over-expression in C2C12 cells results in inhibition of terminal muscle differentiation; there is some controversy about whether this is achieved via direct activation of Notch signalling, as reported by Sakamoto et al. [51], or not [52]. We have found that over-expression of *Nov *pushes 10T1/2 mesenchymal cells down the myogenic pathway, promoting the proliferation and survival of cells expressing myogenin, but blocking terminal differentiation, thus resulting in expansion of the myogenic population (EH, DT, EA, CB personal observations). The muscle cells present in Wilms' tumours exhibiting heterotypic differentiation express a high level of *Nov *[29], consistent with the hypothesis that inappropriate expression of *Nov *may contribute to the abnormal differentiation of mesodermal cells to muscle in some Wilms' tumours. Elevated *Nov *expression is found in musculoskeletal tumours, including alveolar rhabdomyosarcomas [31]. These tumours are thought to originate from a multipotential mesenchymal cell type and are correlated with translocations between chromosomes 1 or 2 and chromosome 13, resulting in the generation of Pax3- or Pax7- forkhead (Pax3-7/FKHR) chimaeric genes [53,54].

There is evidence to suggest that the balance between myogenic and adipogenic potential in myoblasts is regulated by Wnt signalling, with Wnt10b deficiency associated with increased potential for adipogenic differentiation in myoblasts [55]. These authors have proposed that down regulation of Wnt10b signalling may contribute to the impaired muscle regenerative capacity and increased muscle adiposity characteristic of aged muscle. Given that different CCN family members either are induced by Wnts or can themselves modulate Wnt signalling, it would be of interest to determine whether Wnt10b signalling is affected in *Nov*^*del3*^-/- and *Nov*^*del3*^+/- mice.

### Premature cataract formation in *Nov*^*del3 *^homozygotes and heterozygotes

Premature lens degeneration was seen in both *Nov*^*del3*^-/- and *Nov*^*del3*^+/- mice, with early onset cataract formation from six months of age. This was characterised by vacuolation of the lens and loss of the surface epithelium. Cataract formation is very rarely seen in wild type 129/Sv mice of less than twelve months of age, and we found no evidence of this in wild type littermates. Its early onset is also a feature of mice lacking the gap junction protein Connexin 46 (Cx46) [56]. In view of the direct interaction of NOV with Cx43 [47], it would be of interest to determine whether NOV interacts with Cx46 and is required for normal gap junction communication in the lens.

### Nov and maintenance of tissue viability

The cardiomyopathy, muscle atrophy, cataracts and joint abnormalities seen in the *Nov*^*del3*^-/- and *Nov*^*del3*^+/- mice suggest that NOV may have a general role to play in maintenance of tissue viability in adults. This would be consistent with other features of early aging that we have noted in our mice, including hair loss and abnormally low levels of body fat in older animals (EH, CB unpublished observations). NOV has also been implicated in wound healing [40], and has recently been shown to be a key regulator of human haematopoetic stem/progenitor cells [57]. The *Nov*^*del3*^ heterozygous and homozygous mice described here will provide a valuable resource to test the potential involvement of NOV in a variety of processes including stem cell behaviour, tissue regeneration and wound healing.

## Conclusion

There is increasing evidence that CCN family members are important modulators of matricellular signalling in development and disease. In this paper we report the generation of the first mouse mutant in the *Nov *gene; these mice reveal diverse functions for NOV in the embryo and adult and demonstrate for the first time the importance of this protein in organogenesis and in tissue homeostasis and viability in adult mice. We showed that mutation of *Nov *causes overgrowth of the axial and appendicular skeleton, delayed ossification, and severe joint abnormalities. Fibroblasts derived from *Nov*^*del3 *^homozygotes are potentiated to differentiate down the osteogenic pathway, showing the importance of NOV in regulating cell fate decisions and differentiation. We showed that normal NOV function is essential for heart development and that its mutation causes cardiomyopathy in adult mice. This is also the first demonstration that NOV is essential for tissue homeostasis, with premature degeneration of specific muscles and lenses in *Nov*^*del3 *^homozygotes and heterozygotes; these mice may thus provide valuable insights into the processes of tissue viability and aging. A key question in development is how multiple signalling pathways are co-ordinated to orchestrate the complex processes of organogenesis in the embryo and tissue homeostasis in the adult. The *Nov*^*del3 *^mutants described here represent a valuable resource for dissecting these processes at the molecular and cellular level, as well as providing a mouse model for studying diseases affecting the heart, skeleton, muscles and lens.

## Methods

### Generation of constructs, targeted ES cell lines and mice

The targeting construct contained 3.0 kb of 5' homology from the *Xba*I site 1.5 kb upstream of the *Nov *start codon to the first *Pst*I site in intron 2. The 3' homology is 3.1 kb from the *Hind*III site in intron 3 to the *Bam*HI site at the 3'end of intron 4. A TkneopolyA cassette was inserted between the two *Nov *arms and a HSVTK negative selection cassette was inserted downstream of the 3' homology. CCB 129Sv ES cells were electroporated and selected with 0.3 mg/ml G418 and 1 mM Gancyclovir. Colonies were screened by PCR and positive targets confirmed by Southern analysis using external probes (Figure 1A). Germline chimaeras were obtained by injecting karyotypically normal targeted cells into C57Bl/6 blastocysts and the targeted line was maintained on a 129Sv background.

### Derivation and culture of primary cells

Primary embryo fibroblasts (PEFs) were isolated from E13.5 embryos obtained from heterozygous matings and genotyped by PCR using yolk sac DNA. For routine cellular maintenance, PEFs were maintained in Dulbecco's modified Eagle's medium (Invitrogen) containing 10% fetal calf serum. To study chondrogenic/osteogenic differentiation, cells were either plated in high density micromass cultures using the technique of Ahrens *et al*. [58], or as monolayer cultures in 6-well plates seeded at a density of 10^5 ^cells/well. Cultures were maintained in Ham's F12 medium (Invitrogen) containing 10% fetal calf serum. PEF cultures were fixed with 4% (w/v) paraformaldehyde and stained overnight with 1% Alcian Blue 8-GX (pH 1.0) to detect cartilage matrix sulfated glucosaminoglycans [59] or assayed for alkaline phosphatase enzymatic activity by incubation with Sigma Fast BCIP/NBT alkaline phosphatase substrate at 37°C for 10 minutes.

### Reverse transcription RT-PCR and In situ hybridisation

Total RNA was extracted from cultured PEFs using the RNeasy kit (Qiagen) according to manufacturer's instructions. RNA was treated with RNAse free DNAse I and reverse-transcribed using Superscript II (Invitrogen). cDNAs were amplified by PCR using the following primers:

*Gapdh *s GCATGGACTGTGGTCATGAG and as CCATCACCATCTTCCAGGAG; *Nov *specific primers: *Nov *exon1 s GATGCCTCTGCCTAGGCTTC and *Nov *exon4 as CACACTGGCGATTCCTGTTG, *Alkaline phosphatase *s CCTGCAGGATCGGAACG and as GACCTGAGCGTTGGTGTTATATGT; *Collagen I s *AGCACCACGGCAGCAGGAGGTTT and as CAGGGTTGCCAGGAGGTCCAACA; *Osteocalcin *s CCAAGCAGGAGGGCAATA and as AGGGCAGCACAGGTCCTAA. RNA *in situ *hybridization was performed on E16.5 embryo cryostat sections as previously described [32].

### Protein isolation and immunoblot analysis

For whole cell protein lysates, cells were lysed with RIPA buffer and protein concentrations determined using the Bradford assay (Sigma). For Western analysis, 10 μg of each protein sample was subjected to SDS-PAGE (12%) and electrotransferred onto Hybond-P nitrocellulose membranes (GE Healthcare). Blots were probed with a rabbit polyclonal anti-NOV antibody 59.3 (1:1000) raised against a c-terminal NOV peptide (CPQNNEAFLQDLELKTS) which recognizes both full-length NOV (40 kDa) and mutant NOV^del3 ^proteins (30 kDa) or anti-α-tubulin antibody (clone B-5-1-2, 1:3000, Sigma). Proteins were visualized with anti-mouse or anti-rabbit IgG horseradish-peroxidase-linked antibodies (1:25,000; Santa Cruz Biotechnologies) using the ECL detection system (GE Bioscience). To confirm equal protein loading of conditioned media samples, parallel gels were run and stained with Coomassie Brilliant Blue.

### Skeletal preparation and histology

Alcian blue and Alizarin red staining of cleared skeletal preparations was performed according to Hogan et al. [60]. For histology, specimens were fixed in 4% (w/v) paraformaldehyde and embedded in paraffin. Deparaffinized sections (5 μm) were stained with Haematoxylin and Eosin, Alcian blue and Nuclear Fast Red or Von Kossa. Immunostaining for PCNA was carried out on deparaffinized sections after antigen retrevial with 0.1 M sodium citrate using a monoclonal anti-PCNA antibody (1:500, sc-56, Santa Cruz Biotechnologies) and staining detected with the Vectastain Elite Mouse IgG ABC Kit (Vector Laboratories) according to the manufacturers' instructions.

## Authors' contributions

EH contributed to the preparation of the manuscript and performed most of the phenotypic characterisation of the *Nov*^*del3 *^mutants. DT carried out a significant amount of initial work on the *Nov*^*del3*^mutants. EA constructed the targeting construct and isolated the targeted ES cell clones. PS was a co-applicant on the Wellcome grant funding EA and was involved in some of the discussions about the work. SF contributed valuable pathology expertise in analysing the phenotype of the *Nov*^*del3*^mutants. CAB was the principal applicant on the Wellcome Trust and BBSRC project grants that funded the work, conceived and directed the project, generated the *Nov*^*del3*^mice and wrote the paper.

## Supplementary Material

Additional file 1 **Expression analysis of *Nov *in wild type and Nov^del3 ^primary embryonic fibroblasts (PEFs)**. Semi-quantitative RT-PCR of full-length *Nov *(FL Nov) and mutant *Nov*^*del3 *^transcripts (Nov^del3^) in PEFs using Nov exon 1 and exon 4 primers. Wild type (+/+) PEFs express full-length *Nov*, whereas Nov^del3 ^-/- PEFs express only *Nov*^*del3 *^transcripts lacking exon 3. Two fold serial dilutions of cDNA were used and normalised to *gapdh*. Click here for file

Additional file 2 **Serial sections of E13.5 wild type and *Nov*^*del3 *^hearts**. Haematoxylin and Eosin staining of transverse serial sections of E13.5 wild type (A-D) and Nov^del3 ^-/- (E-H) embryonic hearts showing abnormal growth and modelling of endocardial cushions (EC) and delay in fusion of the septum (S) in the mutant embryos (Arrow in E-H). Scale bars, 10 μm. Click here for file
