# Supplementary figures and images for: Abnormal skeletal and cardiac development, cardiomyopathy, muscle atrophy and cataracts in mice with a targeted disruption of the Nov (Ccn3) gene
Source: BMC Dev Biol. 2008 Feb 20;8:18. doi: 10.1186/1471-213X-8-18 (PMC2275724; doi:10.1186/1471-213X-8-18)

## Slide 1
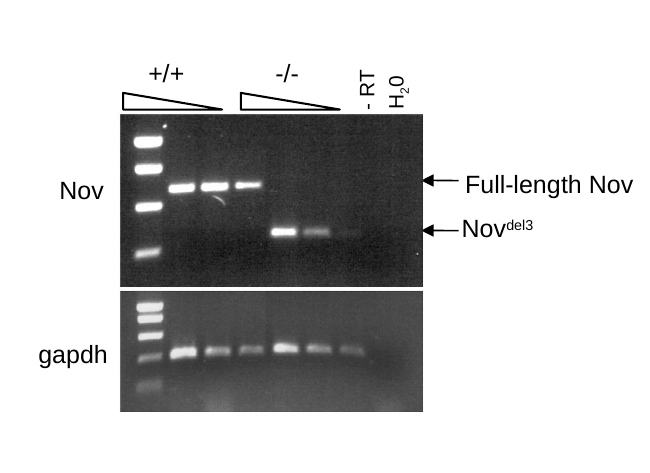

+/+
-/-
- RT
H20
Full-length Nov
Nov
Novdel3
gapdh

Supplement: Additional file 1 — Expression analysis of Nov in wild type and Novdel3 primary embryonic fibroblasts (PEFs). Semi-quantitative RT-PCR of full-length Nov (FL Nov) and mutant Novdel3 transcripts (Novdel3) in PEFs using Nov exon 1 and exon 4 primers. Wild type (+/+) PEFs express full-length Nov, whereas Novdel3 -/- PEFs express only Novdel3 transcripts lacking exon 3. Two fold serial dilutions of cDNA were used and normalised to gapdh. [file 1471-213X-8-18-S1.ppt]

## Slide 1
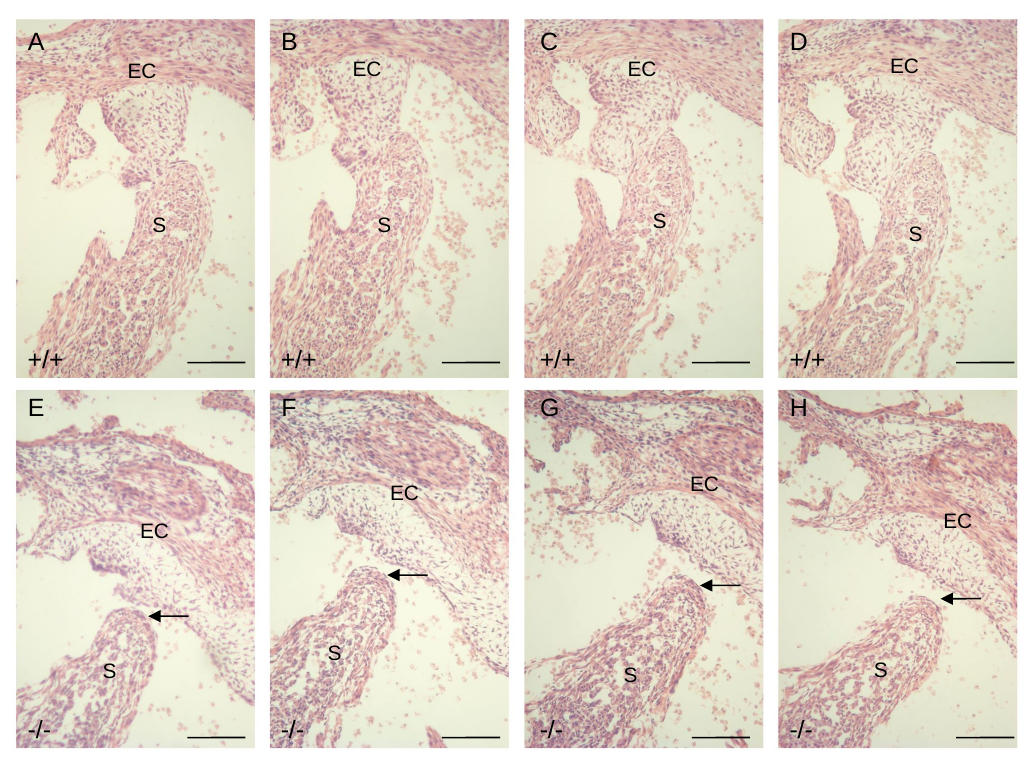

A
B
C
D
EC
EC
EC
EC
S
S
S
S
+/+
+/+
+/+
+/+
E
F
G
H
EC
EC
EC
EC
S
S
S
S
-/-
-/-
-/-
-/-

Supplement: Additional file 2 — Serial sections of E13.5 wild type and Novdel3 hearts. Haematoxylin and Eosin staining of transverse serial sections of E13.5 wild type (A-D) and Novdel3 -/- (E-H) embryonic hearts showing abnormal growth and modelling of endocardial cushions (EC) and delay in fusion of the septum (S) in the mutant embryos (Arrow in E-H). Scale bars, 10 μm. [file 1471-213X-8-18-S2.ppt]
